# Supplementary material for: A history-dependent approach for accurate initial condition estimation in epidemic models
Source: PLoS Comput Biol. 2025 Sep 5;21(9):e1013438. doi: 10.1371/journal.pcbi.1013438 (PMC12445537; doi:10.1371/journal.pcbi.1013438)
Supplement: S1 Text — (DOCX) [file pcbi.1013438.s002.docx]

**S1 Text. Computational package for Hist-D**

We developed a user-friendly computational package, HistD (History-Dependent initial condition decision method), to implement the history-dependent estimation approach in R software version 4.3.2 (https://github.com/Mathbiomed/Hist-D). This package estimates the number of individuals in compartment E by utilizing the data of daily infectious individuals. Although it was developed based on infectious disease models, this method can be generally applied to any compartment model where the time delay between two compartments is known and the count in the latter compartment is available. Detailed instructions for implementation are as follows:

1) Download the package from the code repository (https://github.com/Mathbiomed/Hist-D.git)

2) Save the daily number of infectious people as a CSV file with a name ‘input_data_example.csv’ in the folder containing the main function “HistD_main.R”. Each entry in the first column of ‘input_data_example.csv’ file is the date when the newly infectious people were reported. Each entry in the second column represents the corresponding daily number of people infectious. See the ‘input_data_example.csv’ file for an example of the required format.

3) Open “HistD_main.R”, set the folder containing the R code as a working directory using the function “setwd()”, and compile the essential function library using the R code “source(‘./HistD_source.R’)”.

4) Specify the basic parameters of function “HistD_estimation()”. Assign the initial date ($t_{0}$) of the SEIR model simulation (i.e. the date of starting the simulation) to the “initial_date”. The variable “observed_data” should contain the input data with daily infectious people counts and their corresponding dates (e.g. “input_data_example.csv”) and the variable “total.length” denotes the length of data to be used for the estimation. For example, if the total.length = 5, the HistD_estimation will use the “observed_data” in the dates $t_{0} ,\ldots, t_{0}+4$. The “lambda” parameter controls the magnitude of regularization, with higher values indicating stronger regularization.

5) Specify the distribution type for the latent period by assigning “gamma”, “exponential”, “lognormal”, “weibull” or “empirical” to the variable “dist_type”. If the “dist_type” is “empirical”, add a vector whose $i$-th entry represents the probability of latent period being $i$ days to the variable “discrete_delay”, ensuring that each entry in the vector is greater than zero. For scenarios where delay distributions change at time $t_{0}$, provide the pre-$t_{0}$ and post-$t_{0}$ delay as separate vectors within a list structure to variable “discrete delay”. If the “dist_type” is one of the “gamma”, “exponential”, “lognormal”, or “weibull”, assign the vector with two entries, mean (1st entry) and standard deviation (2nd entry), to the variable “delay_param”. To ensure appropriate values for estimates, provide the mean of the delay distribution to the variable “mean_period”.

6) Execute the code “HistD_estimation()” to obtain the estimated initial conditions from the optimization process. The details of the optimization, such as the formula of loss function, is stated in the code ‘HistD_optim.R’. By revising the lines in the ‘HistD_optim.R’, for example, by revising the ‘HistD_loss’ function, the users can simply customize their loss function. This function will provide the estimated initial condition for compartment E along with the date ($t_{0}$) corresponding to the initial condition for Hist-D method.

7) To assess the uncertainty of our estimation, set the variables to implement “HistD_stan()”. Most variables in this function are common to “HistD_estimation()” function (i.e. the variables “observed_data”, “total.length”, ‘delay_param”, “lambda”, “initial_date”, “dist_type”, and “discrete_delay”, “mean_period”).

8) Specify the additional variables for MCMC. If the variable “stancode” is not explicitly designated, the likelihood of observed data is assumed to follow a Poisson distribution, while other variables counting E and newly exposed individuals follow a Lognormal distribution (See equation (17) in the Methods in the main text for more details). The “stan_code” function in the R file “HistD_interval.R” can be customized based on stan code if different prior distributions are desired. The variable “container” represents the hyperparameters of the prior distribution (i.e. [mean of $k$, standard deviation of $E$, standard deviation of $k$]) as shown in Equation (17) in the Methods section of the main text. The variable “mcmc_param” represents the control options for MCMC (i.e. [chain, iteration, warmup]). Warmup is a burn-in method which incorporates additional tuning for effective sampling.

10) Run the function “HistD_stan()”. The output of “Hist_stan()” function includes the posterior samples of the initial condition (“posterior”), 50%, 75%, 95% credible intervals (“CI”), the point-estimate of initial condition (same with the result from HistD_estimation, “E”) with the date corresponding to the initial condition, and the R-hat statistic from Gelman-Rubin diagnostic test for convergence assessment (“R_hat”). Executing “HistD_stan()” automatically saves the CSV file named “posterior_samples.csv” containing the posterior samples of initial condition of E.

Once all the lines in the “HistD_main.R” are properly implemented, the computation yields point estimates and their corresponding credible interval of the initial condition. Additionally, a CSV file named “posterior_samples.csv” is created. The CSV file contains the posterior samples of the estimated initial values for compartment E. The expected run time depends on scale and the number of data points and the MCMC iterations. For 9 data points ranging from 1000 to 2500 with 100,000 iterations across two chains, the computation time was approximately 27.23 minutes for Hist-D when we tested the example code with MacBook Pro (2023) running macOS Sequoia (15.0.1) with processor M3 11-core with R version 4.4.1 (2024-06-14).
