# Supplementary material for: A history-dependent approach for accurate initial condition estimation in epidemic models
Source: PLoS Comput Biol. 2025 Sep 5;21(9):e1013438. doi: 10.1371/journal.pcbi.1013438 (PMC12445537; doi:10.1371/journal.pcbi.1013438)
Supplement: S2 Text — (DOCX) [file pcbi.1013438.s003.docx]

**S2 Text. Estimation of the reproduction number heavily depends on the initial conditions**

We quantified the impact of the biased initial condition of E on the biased estimation of reproduction number, by employing the simple SEIR model:

|  | $\frac{dS\left( t \right)}{dt}=-\beta\frac{S\left( t \right)I\left( t \right)}{N}$  $\frac{dE\left( t \right)}{dt}=\beta\frac{S\left( t \right)I\left( t \right)}{N}-\kappa E\left( t \right)$  $\frac{dI\left( t \right)}{dt}=\kappa E\left( t \right)-\gamma I\left( t \right)$  $\frac{dR\left( t \right)}{dt}=\gamma I\left( t \right)$ |  |
| --- | --- | --- |

We first simulated this model with initial conditions $S\left( 0 \right)=1000-E\left( 0 \right)-I\left( 0 \right)-R(0), E\left( 0 \right)=50, I\left( 0 \right)=20, R\left( 0 \right)=0$ and $\beta=0.4, \kappa=0.2, \gamma=0.2$. Using the simulated trajectories of S, E , I, and R as data, we fit the model and estimated the transmission rate $\beta$ and the reproduction number $\mathfrak{R} \left( =\beta*\frac{1}{\gamma} \right)$, with different values of $E(0)$ (i.e. we increased or decreased the $E(0)$ from $50$ to $E_{1}$. Specifically, $\log_{2} (E_{1}/50)$ was incrementally increased from -2.5 to 2.5 in steps of 0.5. As a result, for $\log_{2} (E_{1}/50)=2.5,$ we obtained a 42.5% lower value of estimated $\mathfrak{R}$, while a 34.1% higher value of estimated $\mathfrak{R}$ was observed when $\log_{2} (E_{1}/50)=-2.5$, if the true $\mathfrak{R}$ is 4 (S1 Fig). If the true $\mathfrak{R}$ is 2, we observed a 37.9% lower value of estimated $\mathfrak{R}$ when $\log_{2} (E_{1}/50)=2.5$, while $\log_{2} (E_{1}/50)=-2.5$led to a 19.4% higher value of estimated $\mathfrak{R}$.

To investigate how this bias in the initial condition of E evolves in estimates of time-varying reproduction number, $\mathfrak{R}\left( t \right)$ (S1 Fig), we fitted the model to the simulated trajectory of R used in S1 Fig, a, estimating the transmission rate ($\beta\left( t \right)$) and subsequently calculated the reproduction number ($\mathfrak{R}\left( t \right)=\frac{\beta\left( t \right)}{\gamma}\cdot\frac{S\left( t \right)}{N}$). Specifically, if we estimated $\mathfrak{R}\left( t \right)$ at time $t$, we fitted the model to data from 10 days before $t$ (i.e., from $t-10$ to $t$) by minimizing the discrepancy between model predictions and simulated data. This procedure was repeated using different values for the initial condition of $E$: $\frac{E\left( 0 \right)}{4},\frac{E\left( 0 \right)}{2}, E\left( 0 \right), 2E\left( 0 \right),$ and $4E\left( 0 \right)$, where $E\left( 0 \right)$ denotes the true initial value used in generating the synthetic data. The results indicate that the effect of bias in $E\left( 0 \right)$ continues for an extended duration after the time point where the initial condition was estimated and gradually diminishes over time.
