# Supplementary material for: A history-dependent approach for accurate initial condition estimation in epidemic models
Source: PLoS Comput Biol. 2025 Sep 5;21(9):e1013438. doi: 10.1371/journal.pcbi.1013438 (PMC12445537; doi:10.1371/journal.pcbi.1013438)
Supplement: S3 Text — (DOCX) [file pcbi.1013438.s004.docx]

**S3 Text. Hist-D is more accurate than Hist-I, even after Hist-I was modified.**

A recent study by Rauch et al. (1) suggested the modification of Hist-I: summing up the future daily infectious people to estimate the initial condition of E:

$$E\left( t_{0} \right)=\sum_{j=0}^{\tau_{L}-1} f_{E\to I}\left( t_{0}+j \right)$$

We tested this method on the data used in Fig 3c-e and compared it with Hist-D. As a result, Hist-D still estimated more accurately than Hist-I even after the modification (S2 Fig). In particular, Hist-D achieved 80% lower RMSE and 53% lower MAPE than the modified Hist-I, respectively.

**Supplementary References**

1. Rauch W, Schenk H, Rauch N, Harders M, Oberacher H, Insam H, et al. Estimating actual SARS-CoV-2 infections from secondary data. Sci Rep. 2024;14(1):6732.
