# Supplementary material for: A history-dependent approach for accurate initial condition estimation in epidemic models
Source: PLoS Comput Biol. 2025 Sep 5;21(9):e1013438. doi: 10.1371/journal.pcbi.1013438 (PMC12445537; doi:10.1371/journal.pcbi.1013438)
Supplement: S4 Text — (DOCX) [file pcbi.1013438.s005.docx]

**S4 Text. Hist-D enhances the accuracy of reproduction number estimation**

The superior performance of Hist-D over Hist-I motivated us to examine whether this advantage can improve the accuracy of reproduction number ($\mathfrak{R)}$ estimation by providing a more accurate initial condition of E. To do this, we used IONISE, the Bayesian inference method developed by Hong et al. (1) that estimates the reproduction number by adopting history-dependent epidemic dynamics (S4 Fig). Specifically, we applied IONISE to 30-day segments from the simulated R trajectory of synthetic data presented in Fig 3c, starting from various time points ($t=1, 11, 21, \ldots, 161$). For each segment, we estimated the initial condition of E using either Hist-D or Hist-I and incorporated it into the reproduction number estimation of IONISE.

As a result, IONISE combined with Hist-D consistently produced more accurate estimates of $\mathfrak{R}$, than when using Hist-I. In particular, using Hist-I overestimated or underestimated $\mathfrak{R}$ during the increasing phase ($t=0-140$ in Fig 3c) or decreasing phase ($t=150-160$ in Fig 3c) of the epidemic wave, respectively.

**Supplementary References**

1. Hong H, Eom E, Lee H, Choi S, Choi B, Kim JK. Overcoming bias in estimating epidemiological parameters with realistic history-dependent disease spread dynamics. Nat Commun. 2024;15(1):8734.
