# Supplementary material for: A history-dependent approach for accurate initial condition estimation in epidemic models
Source: PLoS Comput Biol. 2025 Sep 5;21(9):e1013438. doi: 10.1371/journal.pcbi.1013438 (PMC12445537; doi:10.1371/journal.pcbi.1013438)
Supplement: S1 Table — The table shows the reduction in RMSE (former) and MAPE (latter) achieved by Hist-D relative to Hist-I under the same conditions as Fig 3c–3e, but with varied latent and infectious period parameters. (DOCX) [file pcbi.1013438.s010.docx]

**Supplementary Tables**

|  | | Infectious period | | |
| --- | --- | --- | --- | --- |
|  |  | (1.25, 4.8) | (2, 3) | (5, 1.2) |
| Latent period | (2, 2.5) | 79.1%, 77.3% | 79.2%, 77.1% | 79.4%, 76.9% |
|  | (5, 1) | 89.1%, 87.2% | 89.2%, 87.2% | 89.3%, 87.4% |
|  | (10, 0.5) | 93.7%, 92.3% | 93.8%, 92.3% | 93.9%, 92.5% |

**S1 Table. Hist-D is more accurate than Hist-I under various parameter conditions.** The table shows the reduction in RMSE (former) and MAPE (latter) achieved by Hist-D relative to Hist-I under the same conditions as Fig 3c–e, but with varied latent and infectious period parameters.
