# Supplementary material for: A history-dependent approach for accurate initial condition estimation in epidemic models
Source: PLoS Comput Biol. 2025 Sep 5;21(9):e1013438. doi: 10.1371/journal.pcbi.1013438 (PMC12445537; doi:10.1371/journal.pcbi.1013438)
Supplement: Attachment — Submitted filename: review.pdf [file pcbi.1013438.s011.pdf]

Lim and colleagues present a method for estimating initial conditions in a generalised SEIR model. Overall, the work appears sound, although I have several concerns for the authors to address as outlined below:

Main comments:

1. General framing: more clarity is needed throughout that the focus is specifically on initial condition estimation in SEIR models with non-exponentially distributed waiting times. If you are using a standard SEIR model with exponentially distributed latent/infectious periods, the "conventional" Hist-I approach would at least be consistent with the underlying model assumptions (if those assumptions are unrealistic, the issue is arguably with the entire model choice rather than the method of estimating initial conditions). Moreover, I am unsure that using the Hist-I approach to estimate initial conditions in SEIR models with non-exponentially distributed wating times is indeed "conventional" – please either provide references to establish this in the introduction, or tone down wording accordingly.
2. Methods: I am unsure about the assumption of a constant rate of exposure before the time at which initial conditions are estimated, and similarly about the choice to minimise the second derivative of daily exposures. These seem questionable in the (likely) scenario where a model is initialised near the start of an epidemic wave, when the outbreak is growing exponentially. At a minimum, I would emphasise these assumptions in the limitations part of the Discussion. I would also be very interested to see results with the relevant assumptions modified to ones consistent with exponential growth, but this is optional (from limited thought, it seems to me potentially doable, if not entirely straightforward).
3. Results: more could be done to establish the consequences of inaccurate estimated initial conditions obtained using Hist-I or (to a lesser extent) Hist-D. Specifically, I would be interested to see a figure like Fig S1 but with points added showing the R estimates that would be obtained with initial conditions estimated using the two methods (perhaps this could be done at a few different time points).
4. I note the manuscript does not currently mention the method of stages, which is probably the most common way of incorporating gamma-distributed waiting times into SEIR models (e.g. https://doi.org/10.1371/journal.pmed.0020174). It seems possible that an alternative method for estimating initial conditions based on an SEIR model with multiple latent substages could be derived. This would be interesting to explore in detail – this is again optional, but at a minimum please at least mention the method of stages in the discussion.

Other points:

1. Abstract/author summary: please make it clear that by "initial" you mean at a pre-selected time of model initiation, rather than at the actual start of the outbreak (e.g. you could write "the initial number of exposed individuals (E) **at the time data first become available**" in line 32).

2. Abstract/author summary: it would be nice to mention explicitly that the Seoul COVID-19 data included likely infection dates, allowing you to directly compare estimated initial conditions with values based directly on the data.
3. Lines 76-77: should “daily infectious people” really be “daily newly infectious individuals” or equivalent (I would interpret the current wording to mean the total number of active cases, rather than the number of new cases)?
4. Line 95: given the emphasis on other people using the provided code, I would like to see it slightly more extensively documented (at a minimum, adding a brief description to each function definition of what that function does, and providing instructions in README.md regarding which script(s) to run to reproduce the results from the paper).
5. Lines 101-102: please make it clear that these results only hold for a specific parameter set.
6. Lines 188-189: I would be interested to see a version of Fig 3c that is zoomed into the early growth phase of the outbreak (perhaps going back before time 80), given this is when a model would most likely be initialised (but you can’t really see how the methods in this phase in the current figure).
7. Lines 191-194: relatedly, in Fig S2, does the modified Hist-I outperform Hist-D either in the earliest or latest stages of the outbreak (it seemingly does in one of the two, but I’m not sure which)? If the former, this should be mentioned.
8. Lines 262-270: it may be worth saying something here about models distinguishing infectiousness from symptoms/reporting, given that you equate the two here but for COVID-19 the former typically precedes the latter.
9. Lines 268-270: I’m not sure why the conclusion appears halfway through the Discussion section – please check and amend/reorder as appropriate.
10. Lines 326-335: do the $\tilde{f}$ variables represent rates rather than numbers (I am not sure the latter makes sense in a continuous-time model)?
11. Lines 408-409: the second line of equation (18) looks potentially incorrect to me – please check.
12. Caption to Fig S1: should (e.g.) $\log_2(E_1/E_0) = 2^{2.5}$ be $E_1/E_0 = 2^{2.5}$ (or equivalently, $\log_2(E_1/E_0) = 2.5$)?
13. Lines 424-425: is it possible to produce a plot comparing the incidence data in Fig. 4a with an equivalent version using all notified cases (not just those with available contact and symptom onset dates), to confirm there’s no substantial bias arising through a time-varying rate of contact/symptom data collection?
14. Lines 429-430: how did you calculate the value of E at the first time point?
