# Supplementary material for: A history-dependent approach for accurate initial condition estimation in epidemic models
Source: PLoS Comput Biol. 2025 Sep 5;21(9):e1013438. doi: 10.1371/journal.pcbi.1013438 (PMC12445537; doi:10.1371/journal.pcbi.1013438)
Supplement: Attachment — Submitted filename: PLoS Comp Biol_review_3-25.pdf [file pcbi.1013438.s012.pdf]

Review of

"A history-dependent approach for accurate initial condition estimation in epidemic models" by D. Lim et al.

March 2025

Overview

This is an interesting paper that introduces a new method for estimating the initial condition of the exposed (E) class in an SEIR epidemic model that considers exposure history. Conventional methods do not account for different timing of exposure among individulas in compartment E, and the main results of this paper compare the improved accuracy of the history-dependent method to the conventional (history-independent) method. The authors apply their method to COVID-19 data from Seoul, South Korea during the first year of the pandemic, and they show that their method significantly reduces the estimation error compared to the conventional method. This is an original and important contribution to the field and the methodology is rigorously developed. The authors also provide a well documented R package to run simulations and to facilitate the use of their method. The manuscript is well written and well-organized. I only have minor comments and a few typos to fix. I recommend publication with minor revision, as detailed below.

Comments

My main comment is about the motivation for why the authors consider a history-dependent approach for initial condition estimation. Specifically, the first paragraph of the Results section could be expanded to include more detail and the motivation could be made more clear. One idea is to move or summarize some of the information from the Supplement (Fig 1 and Note 2) to this paragraph. Or a small table in the main text here could give a better interpretation of Supplementary Fig 1 and Note 2 for a few different scenarios (% reduced or increased initial condition and resulting error). While reading this first paragrah in the Results, I found myself paging back through the Supplementary info and it broke up the flow of the paper for me. Adding a little more motivating details and justification here will greatly improve readability. Also, it wasn't clear where the 33.9% relative error cited in this paragraph came from... it wasn't easy to quickly skim the Supplementary info and figure that out. Consider making this easier to decipher for the reader.

Another main comment is that in the abstract, the authors say that applying their method to COVID-19 data from Seoul results in reducing the estimation error by 55% compared to the conventional method. However, there are two separate instances of this same claim in the text (lines 92 and 259) but the reduction in the error says 40%. I believe this is a typo. Indeed, the second one refers to Fig 4 which also seems to agree with the 55% claim. If these are not typos, please clarify why these are different.

Minor Comments / Typos

- Line 106: Fig 1b is mentioned first, but Fig 1a is left out of the main text discussion. This could go along with the expansion of this paragraph as described above, but at the very least consider a brief mention of Fig 1a and how to interpret the insert in the figure before discussing Fig 1b.

- Line 192: Add a brief summary of modifications made. This is easier for the reader than going to the supplementary info. Then, if the reader is interested they can read further.

- Line 195: "Superior accuracy" - this is vague, consider adding by how much or by what range.

- Lines 196-196: What exactly do you mean by ideal conditions? No measurement error? Please add a brief statement here.

- Lines 203-204: Why did you choose noise levels up to 0.1? Add a justification or comment here.

- Lines 226-227: Consider a comment on whether the distribution of the latent period in this data looks gamma distributed.

- Line 350: Please comment on why this is a reasonable assumption for the approximation.

- Lines 405-406: Please justify or comment on why you used the parameters from Refs 19 and 28.

- Line 447: typo - extra comma after $\mu_k =$

- Line 471: typo - "The codes underlying this work are..."
