# Supplementary material for: A history-dependent approach for accurate initial condition estimation in epidemic models
Source: PLoS Comput Biol. 2025 Sep 5;21(9):e1013438. doi: 10.1371/journal.pcbi.1013438 (PMC12445537; doi:10.1371/journal.pcbi.1013438)
Supplement: Attachment — Submitted filename: Response to reviewers.docx [file pcbi.1013438.s014.docx]

We thank the reviewers for their thorough and constructive comments. In light of these comments, we have made substantial revisions to our manuscript, resulting in a significant enhancement of its readability and impact.

Below, you will find a point-by-point response to each comment. Our responses are indicated in blue, while the revised portions of the manuscript are highlighted in green (with italic font indicating the modified section).

**Editor:**

Thank you for sending this very nice paper to PLOS Computational Biology. I was fortunate to receive comments from three reviewers, all of whom have engaged with the paper and provided useful and relevant comments. All three agree with my own view that this is an interesting and potentially important contribution, considering how the initial number of latently infected individuals can be better estimated from data, going beyond a broad brush assumption based on the initial infected population and the expected latent period. However, in different ways all reviewers comment on how the work could be slightly better motivated and/or generalised: I was struck by R3's comment about transmission before symptoms, which is common for pathogens of a range of host taxa, and this should at least be discussed. I was also struck by R1's comments around making the case more strongly that the so-called Hist-I assumption is, in fact, "conventional"/widely used, and agree that no consideration of the method of stages is surprising. While these issues can, probably, be handled by adding more text, some of the other more detailed issues raised by R3 in their review might require additional computational work/reworking of some figures. I will also be very interested to read the authors' response to R3's comment around the potential for error introduced by assuming a constant rate of exposure for an epidemic that is growing exponentially, and it might be that some further tests with synthetic data might be helpful here. Both R1 and R3 raise minor issues with the supplied code or its documentation, and for this type of article I think these are important to address in full. Nevertheless, this appears at this stage to be a very nice article, and I look forward to seeing a revision, which at this stage I intend to send to (at least) R1 again (perhaps also R2 and R3, depending on what is said in the response to reviewers letter).

Journal requirements:

1. Please ensure that the CRediT author contributions listed for every co-author are completed accurately and in full. At this stage, the following Authors/Authors require contributions: Dongju Lim, Kyeong Tae Ko, Hyojung Lee, Boseung Choi, Won Chang, Sunhwa Choi, and Jae Kyoung Kim. Please ensure that the full contributions of each author are acknowledged in the "Add/Edit/Remove Authors" section of our submission form.
   The list of CRediT author contributions may be found here: <https://journals.plos.org/ploscompbiol/s/authorship#loc-author-contributions>
2. Please upload all main figures as separate Figure files in .tif or .eps format. For more information about how to convert and format your figure files please see our guidelines:
   <https://journals.plos.org/ploscompbiol/s/figures>
3. We have noticed that you have uploaded Supporting Information files, but you have not included a complete list of legends. Please add a full list of legends for your Supporting Information files (HIstD.zip) after the references list.
4. In the online submission form you indicate that your data is not available for proprietary reasons and have provided a contact point for accessing this data. Please note that your current contact point is a co-author on this manuscript. According to our Data Policy, the contact point must not be an author on the manuscript and must be an institutional contact, ideally not an individual. Please revise your data statement to a non-author institutional point of contact, such as a data access or ethics committee, and send this to us via return email. Please also include contact information for the third party organization, and please include the full citation of where the data can be found.
5. Please amend your detailed Financial Disclosure statement. This is published with the article. It must therefore be completed in full sentences and contain the exact wording you wish to be published.
6. State what role the funders took in the study. If the funders had no role in your study, please state: "The funders had no role in study design, data collection and analysis, decision to publish, or preparation of the manuscript."

We thank the editor for the positive comment. We have revised our manuscript to comply with the journal’s formatting requirements. Specifically, we have updated the author contributions, provided figures in EPS format, added a complete list of legends for the supporting information, included contact information for the data provider, and revised the financial disclosure statement. However, we were unable to provide a full citation for the data, as it is not publicly available.

**Reviewer #1:**

Lim and colleagues present a method for estimating initial conditions in a generalised SEIR model. Overall, the work appears sound, although I have several concerns for the authors to address as outlined below:

Major:

1. General framing: more clarity is needed throughout that the focus is specifically on initial condition estimation in SEIR models with non-exponentially distributed waiting times. If you are using a standard SEIR model with exponentially distributed latent/infectious periods, the “conventional” Hist-I approach would at least be consistent with the underlying model assumptions (if those assumptions are unrealistic, the issue is arguably with the entire model choice rather than the method of estimating initial conditions). Moreover, I am unsure that using the Hist-I approach to estimate initial conditions in SEIR models with non-exponentially distributed waiting times is indeed “conventional” – please either provide references to establish this in the introduction, or tone down wording accordingly.

We thank the reviewer for this valuable comment. As the reviewer pointed out, our manuscript focuses on the SEIR model with non-exponentially distributed waiting times. While this history-dependent SEIR model has been extensively studied using approaches such as the method of stages, linear chain tricks, and delay differential equation (DDE) models, the estimation of initial conditions for such models has remained unknown. To address this gap, we developed Hist-D, which estimates the initial condition of E within a history-dependent SEIR model context. We have revised the Introduction section to clarify this general framing of the manuscript.

**Introduction:**

**…**

*Relaxing the unrealistic history-independent assumption of a standard SEIR model, requires two components: (i) a model reflecting the changing likelihood of transitioning to the infectious stage, and (ii) accurate initial conditions for such a model. The first component has been extensively studied through approaches such as the method of stages (15), linear chain tricks (16, 17), and delay differential equation (DDE) models (18). Applying this model, particularly the DDE-based model, enabled more accurate estimation of epidemic parameters by incorporating individual exposure history (18). However, despite this advantage, a method for accurately estimating their initial conditions—particularly the number of exposed individuals—remains unknown.*

…

Furthermore, we acknowledge the reviewer’s concern regarding the labeling of Hist-I as a “conventional” method. Rather than implying widespread adoption, we use Hist-I as a method following the fundamental assumption of the history-independent SEIR model (i.e., SEIR model with exponentially distributed waiting times). Specifically, in this model, the daily incidence of becoming infectious ($f_{E\to I}\left( t_{0} \right)$) is the product of exposed populations ($E\left( t_{0} \right)$) and the rate of becoming infectious ($\kappa$), which is the inverse of the average latent period ($\mathbb{E}\left[ \tau_{L} \right]=1/\kappa$). This assumption naturally leads to the equation ($E\left( t_{0} \right)=f_{E\to I}\left( t_{0} \right)\mathbb{\cdot E}\left[ \tau_{L} \right]$), which underpins the Hist-I method described in our manuscript. We have clarified this point in the revised manuscript and revised our language to avoid overgeneralizing the applicability of Hist-I beyond its appropriate context.

**Abstract:**

*…~~Conventionally,~~* This initial condition has *often* been estimated using an unrealistic, history-independent assumption for simplicity:

**Introduction:**

… An alternative approach estimates the initial condition of E using the known number of daily infectious people (14). This approach *is consistent with the fundamental assumption of a standard SEIR model—*the daily number of new infectious people is the product of (i) the population in the exposed compartment and (ii) the rate of progression to the infectious stage, which is reciprocal to the length of the latent period.

**Results:**

**The ~~conventional~~ history-independent method is inaccurate when the latent period is non-exponential**

…

To overcome these limitations and set the initial conditions using the available data, *the standard SEIR model can be used* (Fig 1c; See S2 Text for more details). Specifically, $f_{E\to I}\left( t_{0} \right)$ is the product of exposed populations ($E\left( t_{0} \right)$) and the rate of becoming infectious ($\kappa$), which is the inverse of the average latent period ($\mathbb{E}\left[ \tau_{L} \right]=1/\kappa$). This assumption naturally leads to the equation $E\left( t_{0} \right)=f_{E\to I}\left( t_{0} \right)\mathbb{*E}\left[ \tau_{L} \right]$: the initial condition of E can be estimated by multiplying the average latent period ($\mathbb{E}\left[ \tau_{L} \right]$) by the $f_{E\to I}$ at the initial time point ($f_{E\to I}(t_{0})$) (14).

*This method, History-Independent estimation (Hist-I), follows the core assumption of the standard SEIR model* that does not consider the time when individuals are exposed (i.e. exposure history), assuming everyone experiences the same chance of becoming infectious regardless of their exposure history (Fig 1d).…

2. Methods: I am unsure about the assumption of a constant rate of exposure before the time at which initial conditions are estimated, and similarly about the choice to minimise the second derivative of daily exposures. These seem questionable in the (likely) scenario where a model is initialised near the start of an epidemic wave, when the outbreak is growing exponentially. At a minimum, I would emphasise these assumptions in the limitations part of the Discussion. I would also be very interested to see results with the relevant assumptions modified to ones consistent with exponential growth, but this is optional (from limited thought, it seems to me potentially doable, if not entirely straightforward).

We thank the reviewer for the valuable comment. In our loss function, we adopted a regularization term that minimizes the second derivative of daily exposure—a widely used approach for capturing smooth trends in time-series data (DOI: 10.1016/j.compchemeng.2009.10.007). In addition, we assumed constant daily exposure before the initial time point to simplify the equation. This assumption, as the reviewer pointed out, may not hold near the start of an epidemic wave, when exponential growth is more realistic—as indeed reflected in synthetic data (Time = 80 - 140 in Fig. 3a). However, it is noteworthy that Hist-D accurately estimated the initial condition of E, even in such exponential growth scenarios. This accurate estimation was also evident in the real-world COVID-19 dataset during the exponential growth phase from 2020-09-30 to 2020-11-17 (Fig. 4a).

Nonetheless, to investigate whether relaxing the constant assumption can enhance the accuracy of Hist-D, we developed a modified version, Hist-D-exp, which assumes exponential growth (or decay) in daily exposure before the initial time point. When applied to real-world COVID-19 data, Hist-D-exp produced results merely comparable in accuracy to those of the original Hist-D (Fig. R1).

**Fig. R1 Relaxing the constant exposure reaction does not enhance the performance of Hist-D.** The graph compares the true $E\left( t_{0} \right)$ (light gray bars) and estimated $E\left( t_{0} \right)$ (Hist-I: red triangles, Hist-D: green squares, Hist-D-exp: blue circles). Hist-D and Hist-D-exp yield comparable estimates of $E\left( t_{0} \right)$.

This similarity in performance may stem from the additional parameter required in Hist-D-exp—the rate of exponential growth or decay—which complicates the optimization process. To enhance the parameter estimation process, further refinement of Hist-D-exp is needed, which we consider a promising direction for future work. These points have been added to the revised manuscript, including a discussion of the limitations.

**Discussion:**

Despite the novelty of Hist-D, several limitations should be noted. First, our methods are derived from ODE-based infectious disease models, though PDE-based models are also used to account for spatial effects in disease spread (44, 45). Whether Hist-D can be extended to PDE models remains an open question, and exploring this would be a promising direction for future research. *In addition, Hist-D assumes a constant daily incidence of exposure before the initial time point (See equation (8) in the Methods section). Relaxing this assumption to accommodate scenarios such as super spreading events (46) or exponential growth (or decay) represents another important direction for future work.*

3. Results: more could be done to establish the consequences of inaccurate estimated initial conditions obtained using Hist-I or (to a lesser extent) Hist-D. Specifically, I would be interested to see a figure like Fig S1 but with points added showing the R estimates that would be obtained with initial conditions estimated using the two methods (perhaps this could be done at a few different time points).

Thank you for the valuable suggestion. To examine the impact of initial conditions estimated using Hist-I and Hist-D on reproduction number estimation, we employed IONISE, the Bayesian inference method developed by Hong et al. (ref 18) that estimates the reproduction number by adopting history-dependent epidemic dynamics (S4 Text and S4 Fig). We applied IONISE to the 30-day segments of the synthetic data presented in Fig. 3c, starting from various time points (t = 1, 11, 21, ..., 161). For each segment, the initial condition at the starting point was estimated using either Hist-I or Hist-D and subsequently used in IONISE to estimate the reproduction number.

The results showed that, across all time points, IONISE combined with Hist-D consistently produced more accurate estimates of the reproduction number than when using Hist-I. In particular, Hist-I introduced considerable bias: overestimating the reproduction number during the growing phase (t = 1 - 141 in Fig 3c), underestimating it during the declining phase (t = 151 - 161 in Fig 3c). These findings demonstrate the potential of Hist-D to improve the accuracy of epidemiological parameter estimation. We have included these findings in the revised manuscript.

**Discussion:**

… Nonetheless, the advantage of using history-dependent models relies heavily on accurate initial conditions (*S4 Text and S4 Fig*), as these values significantly affect the model predictions. Previous methods for determining initial conditions were based on a history-independent assumption, misaligning with the dynamics in history-dependent models and resulting in a considerable bias in initial conditions (Fig 3) and subsequent estimation of the reproduction number (*S4 Fig*). We addressed this here by developing a history-dependent method for estimating the initial condition (*S4 Fig*). This, combined with history-dependent models, provides the first framework that completely describes the history-dependent dynamics of infectious disease.

**Supporting information:**

**S4 Text. Hist-D enhances the accuracy of reproduction number estimation**

*The superior performance of Hist-D over Hist-I motivated us to examine whether this advantage can improve the accuracy of reproduction number (*$\mathfrak{R)}$ *estimation by providing a more accurate initial condition of E. To do this, we used IONISE, the Bayesian inference method developed by Hong et al. (2) that estimates the reproduction number by adopting history-dependent epidemic dynamics (S4 Fig). Specifically, we applied IONISE to 30-day segments from the simulated R trajectory of synthetic data presented in Fig 3c, starting from various time points (*$t=1, 11, 21, \ldots, 161$*). For each segment, we estimated the initial condition of E using either Hist-D or Hist-I and incorporated it into the reproduction number estimation of IONISE.*

*As a result, IONISE combined with the Hist-D consistently produced more accurate estimates of* $\mathfrak{R}$*, than when using Hist-I. In particular, using Hist-I overestimated or underestimated* $\mathfrak{R}$ *during the increasing phase (*$t=0-140$ *in Fig 3c) or decreasing phase (*$t=150-160$ *in Fig 3c) of the epidemic wave, respectively.*

***S4 Fig. Hist-D enhances the accuracy of reproduction number estimation.*** *Boxplots of the posterior samples of the reproduction number (*$\hat{\mathfrak{R}}$*) obtained from IONISE with initial conditions estimated by Hist-D (green) and Hist-I (red). IONISE combined with Hist-D accurately estimated the reproduction number, while using Hist-I introduced a considerable bias. Here, the posterior samples were normalized by the true value (*$\mathfrak{R}$*) employed for generating the simulation data depicted in Fig 3c.*

4. I note the manuscript does not currently mention the method of stages, which is probably the most common way of incorporating gamma-distributed waiting times into SEIR models (e.g. https://doi.org/10.1371/journal.pmed.0020174). It seems possible that an alternative method for estimating initial conditions based on an SEIR model with multiple latent substages could be derived. This would be interesting to explore in detail – this is again optional, but at a minimum please at least mention the method of stages in the discussion.

As the reviewer pointed out, gamma-distributed waiting times can be incorporated into the SEIR model using the method of stages, which introduces multiple subclasses within the “Exposed” (E) compartment. However, under this framework, the number of E subclasses becomes a tuning parameter. Determining its optimal value requires evaluating multiple models with varying numbers of subclasses, thereby significantly increasing the computational cost of the optimization process. More importantly, the method of stages constrains the latent period to follow an Erlang distribution (i.e., a gamma distribution with an integer-valued shape parameter). To address these limitations, we adopted an alternative approach based on a previous study (ref. 18), which allows for the incorporation of an arbitrary latent period distribution. We have clarified this point in the Methods section.

**Method:**

We established the total loss function to estimate the initial conditions of exposed individuals. This loss function started from the master equation *that characterizes the history-dependent rate of becoming infectious by incorporating a non-exponentially distributed latent period. While this can be modeled through the method of stages (15), which introduces multiple substages in the exposed compartment, it requires specifying the number of substages as an additional tuning parameter. Identifying its optimal value requires evaluating multiple models with varying numbers of substages, thereby significantly increasing the computational cost of the optimization process. More importantly, the method of stages constrains the latent period to an Erlang distribution. To avoid this, we adopted an alternative approach, following a previous study (18),* which allowed us to incorporate an arbitrary latent period distribution. In particular, the instantaneous rate of individuals becoming infectious is a convolution of the instantaneous rate of individuals exposed and the probability density function of the latent period, as follows (Fig 2b (i)):

Minor:

1. Abstract/author summary: please make it clear that by “initial” you mean at a pre-selected time of model initiation, rather than at the actual start of the outbreak (e.g. you could write “the initial number of exposed individuals (E) at the time data first become available” in line 32).

We have clarified the meaning of “initial” in the Abstract/Author summary as suggested.

**Abstract:**

…In infectious disease models, for instance, the initial number of exposed individuals (E) *at the time the model simulation starts* is frequently unknown…

**Author summary:**

Accurately predicting infectious disease spread requires knowing *the initial number of individuals in the exposed compartment at the start of the simulation* (E(t0)), but this number is usually unknown.

2. Abstract/author summary: it would be nice to mention explicitly that the Seoul COVID-19 data included likely infection dates, allowing you to directly compare estimated initial conditions with values based directly on the data.

Thank you for the suggestion. We have revised the sentences to include additional details about the data.

**Abstract:**

…In particular, our method *reduced estimation error by 55% compared to the previous method* in real-world COVID-19 data from Seoul, Republic of Korea, *which includes likely infection dates, allowing us to obtain the true initial condition*….

**Author summary:**

….In particular, our approach accurately estimated E(t0) for COVID-19 data from Seoul *that includes likely infection dates, which allowed us to obtain the true initial condition*…

3. Lines 76-77: should “daily infectious people” really be “daily newly infectious individuals” or equivalent (I would interpret the current wording to mean the total number of active cases, rather than the number of new cases)?

Thank you for pointing this out. As the reviewer correctly pointed out, the term “daily infectious people” represents the the daily number of individuals who newly become infectious. To avoid the possible confusion, we have revised “daily infectious people” to “daily incidence of becoming infectious” and “daily exposed people” to “daily incidence of exposure” throughout the manuscript.

4. Line 95: given the emphasis on other people using the provided code, I would like to see it slightly more extensively documented (at a minimum, adding a brief description to each function definition of what that function does, and providing instructions in README.md regarding which script(s) to run to reproduce the results from the paper).

Thank you for your comment. We added more description to the main functions in our Hist-D package and provided the code needed to reproduce the results in the manuscript.

**README.md:**

Hist-D

*This repository provides simulation data to reproduce the results shown in Figure3.*

1. *Inference in original simulation data (Fig. 3c ~ 3e).*
   a. *Run “Figure3_cde.R” file.*
2. *Inference in simulation data with measurement errors (Fig. 3f ~ 3h).*
   a. *Run “Figure3_fg.R” and “Figure3_h.R” file.*
3. *Inference in simulation data when transmission rate changes (Fig. 3i ~ 3k).*
   a. *Run “Figure3_ijk.R” file.*

 Main functions

-  	*HistD_estimation : Function to estimate the initial condition using the Hist-D method (See detailed description of options in supporting information). This function returns the estimated initial condition projected for the specified date.*

-  	*HistD_stan : Function to calculate the credible interval of the initial condition using the MCMC method (See detailed description of options in supporting information). These results contain 50%, 75% and 95% credible intervals of the initial condition and R hat for convergence diagnostics of the MCMC chains.*

*Simulation data*

*You can construct the simulation SEIR data using delay differential equations (DDE) with the “mean_trajectory_SEIR_original()” function in the “SEIR_function.R” file.*

5. Lines 101-102: please make it clear that these results only hold for a specific parameter set.

We have clarified that the parameter condition used here mimics that of COVID-19, consistent with the parameter setting applied in Fig 3c–e.

**Results:**

… For example, if the initial condition for the exposed population is reduced to 25% of the original value, this leads to a 33.9% relative error in estimating the reproduction number *under the parameter condition mimicking the COVID-19 dynamics*,…

6. Lines 188-189: I would be interested to see a version of Fig 3c that is zoomed into the early growth phase of the outbreak (perhaps going back before time 80), given this is when a model would most likely be initialised (but you can’t really see how the methods in this phase in the current figure).

As suggested by the reviewer, we investigated the performance of Hist-D and Hist-I during the early phase of the outbreak (i.e., before time 80; S3 Fig). As a result, Hist-D consistently outperformed Hist-I, reducing both the root mean squared error (RMSE) and mean absolute percentage error (MAPE) by 81.0%. This result has been added as S3 Fig in the revised manuscript.

**Results:**

… As a result, Hist-D was much more accurate than Hist-I (Fig 3d), particularly reducing the root mean squared error (RMSE) and mean absolute percentage error (MAPE) by 85% and 84%, respectively (Fig 3e). *Similar improvements were also observed during the earlier phase (*$t_{0}=10, \ldots, 80$*) of epidemic growth (S3 Fig).*

**Supporting information:**

***S3 Fig. Hist-D outperforms both Hist-I and the modified Hist-I even in the early phase of the epidemic dynamics.*** *(a) The graph comparing the true* $E(t_{0})$ *(light gray-colored bars) and the estimated* $E(t_{0})$ *(*$\hat{E}(t_{0})$*) for time* $t_{0}=10-80$*. (b) The scatter plot displaying the error between estimated* $E\left( t_{0} \right)$ *(*$\hat{E}\left( t_{0} \right)$*) and true* $E\left( t_{0} \right)$ *across different levels of true* $E\left( t_{0} \right)$*. Estimation from Hist-D (green squares) shows smaller errors compared to both Hist-I (red triangles) and modified Hist-I (blue circles). (c)* *The bar plot showing the root mean squared error (RMSE; bars) and mean absolute percentage error (MAPE; line) of Hist-I, the modified Hist-I, and Hist-D. Hist-D reduced both RMSE and MAPE by 81% compared to Hist-I, whereas the modified Hist-I reduced both by 47%.*

7. Lines 191-194: relatedly, in Fig S2, does the modified Hist-I outperform Hist-D either in the earliest or latest stages of the outbreak (it seemingly does in one of the two, but I’m not sure which)? If the former, this should be mentioned.

In the earliest stage of the outbreak (t = 10 - 80 in Fig 3c), Hist-D consistently outperforms the modified Hist-I (S3 Fig and Fig. R2a, e). Although the modified Hist-I showed better performance than Hist-D during the latest stage (t = 190 - 210 in Fig 3c; Fig. R2b, f), we found that a slight change in the latent period distribution (i.e., from $\Gamma(4.06, 1.35)$ to $\Gamma(4, 1.4)$) reversed this result, with Hist-D outperforming the modified Hist-I (Fig. R2d, h). This indicates that the superior performance of the modified Hist-I in the latest stage is highly sensitive to latent period assumptions. In contrast, Hist-D outperformed the modified Hist-I under both latent period conditions during the earliest stage (Fig. R2c, g), when model initialization is most likely to happen. Taken together, these findings suggest the superiority of Hist-D over the modified Hist-I, and thus, we have limited our discussion of the modified Hist-I to its current scope in the manuscript.

**Fig. R2 Hist-D outperforms the modified Hist-I in the earliest stage of the outbreak.** (a-b) The graph comparing the true $E\left( t_{0} \right)$ (light gray-colored bars) and the estimated $E\left( t_{0} \right)$ ($\hat{E}\left( t_{0} \right)$) during the earlier stage (a) or later stage (b) with the latent period following $\Gamma\left( 4.06, 1.35 \right)$. Hist-D (green squares) outperforms the modified Hist-I (blue triangles) in the earlier stage, while the result was reversed in the later stage. (c-d) Same plot as (a-b) but with the latent period following $\Gamma\left( 4, 1.4 \right)$. Hist-D outperforms the modified Hist-I in both the earlier stage and the later stage. (e-f) The scatter plot displaying the absolute error between estimated $E\left( t_{0} \right)$ ($\hat{E}\left( t_{0} \right)$) and true $E\left( t_{0} \right)$ across different levels of true $E\left( t_{0} \right)$ during the earlier stage (e) or later stage (f) with the latent period following $\Gamma\left( 4.06, 1.35 \right)$ (g-h) Same plots as (e-f), but with the latent period following $\Gamma\left( 4, 1.4 \right)$.

8. Lines 262-270: it may be worth saying something here about models distinguishing infectiousness from symptoms/reporting, given that you equate the two here but for COVID-19 the former typically precedes the latter.

We thank the reviewer for this valuable comment. As recommended, we have discussed models distinguishing infectiousness from symptoms/reporting.

**Discussion:**

… *This flexibility allows* Hist-D *to* be *applied* to other infectious disease models, such as SEIR-Vaccinated (SEIRV) (28, 29), SEI-Quarantined-R (SEIQR) (30, 31), or *SE-Presymptomatic-IR (SEPIR) (32, 33)*. For more complex models (15) with additional divisions in the exposed (E) or infectious (I) compartments, the same approach used in Hist-D can be adapted by modifying the left and right sides of the equations derived in this study…

However, we would like to clarify that we did not equate infectiousness with symptom/reporting, and we sincerely apologize for any confusion caused by our use of the term “latent period” in the COVID-19 data analysis. Rather than “latent period”, a more accurate term would have been “incubation period”, as we calculated the transition time from exposure to symptom onset. Importantly, this correction does not affect the applicability or results of Hist-D, which remains valid in any setting where the transition time between two compartments (e.g., latent period) is known and inflow data for the downstream compartment (e.g., daily incidence of becoming infectious) is available. We have clarified this point in the revised manuscript and replaced the term “latent period” with “incubation period” in the fourth section of the Results section.

**Discussion:**

…

 Although this study focused on the SEIR model, *Hist-D can be applied to any compartmental model where the transition time between two compartments is known and inflow data for the downstream compartment is available. Thus, Hist-D can be used when the daily incidence of becoming infectious and the latent period distribution are known (Fig 3), or when the daily incidence of symptom onset and the incubation period distribution are known (Fig 4). This flexibility allows* Hist-D *to* be *applied* to other infectious disease models, such as SEIR-Vaccinated (SEIRV) (28, 29), SEI-Quarantined-R (SEIQR) (30, 31), *or SE-Presymptomatic-IR (SEPIR) (32, 33)*. For more complex models (15) with additional substages in the exposed (E) or infectious (I) compartments, the same approach used in Hist-D can be adapted by modifying the left and right sides of the equations derived in this study (see Fig 2b and ‘Derivation of loss function’ section of Method for more details). …

**Results:**

**Hist-D outperforms the Hist-I for real-world COVID-19 data**

… To test this, we applied Hist-I and Hist-D to the reported COVID-19 cases data from Seoul, Republic of Korea, spanning August 13^th^ to November 25^th^, 2020. This data included the contact dates and symptom onset for people in Seoul, allowing us to empirically derive the number of people moving from S to E ($f_{S\to E}$) and from E to I ($f_{E\to I}$), as well as the distribution of the *incubation* period (i.e., the time between contact and symptom onset date) (See Methods for more details) (Fig 4a). …

9. Lines 268-270: I’m not sure why the conclusion appears halfway through the Discussion section – please check and amend/reorder as appropriate.

As the reviewer pointed out, the corresponding sentence was inappropriate. We have revised the sentence.

**Discussion:**

…These modifications can also be easily implemented in our Hist-D package by altering a single function (see (7) of the S1 Text for further guidance). *Therefore, Hist-D offers a flexible framework that can be readily extended, and applying it to a broader class of infectious disease models represents a promising direction for future research*

10. Lines 326-335: do the 𝑓̃ variables represent rates rather than numbers (I am not sure the latter makes sense in a continuous-time model)?

The $\tilde{f}$ variables, $\tilde{f}_{S\to E}$ and $\tilde{f}_{E\to I}$, represent transition rates in continuous time, whereas the $f$ variables, $f_{S\to E}$ and $f_{E\to I}$, denote discretized counterparts. This distinction is necessary to reconcile the continuous-time framework of the model with the discrete-time resolution of real-world data. For example, the input data used in Hist-D records the number of individuals who transition from exposed to infectious over each one-day interval, and is therefore collected on a daily basis. In contrast, the corresponding term in the mathematical model, $Flow_{E\to I}$ (i.e., $\tilde{f}_{E\to I}$), represents the instantaneous rate at a specific time point (See Eq. (16) in the Methods). To reconcile this discrepancy, we integrated $\tilde{f}_{E\to I}$ over time to define $f_{E\to I}$ (See Eq. (2) in the Methods). We also discretized $\tilde{f}_{S\to E}$ ($Flow_{S\to E}$ in Eq. (16)) to ensure consistency between $f_{S\to E}$ and $f_{E\to I}$—specifically, to align the time points when both transitions were measured. This allowed us to construct the matrix equation (Fig 2c), which was then used to formulate the loss function. To clarify the distinction, we have explicitly stated that $\tilde{f}$ variables represent instantaneous rates

**Methods:**

**Derivation of the total loss function used in Hist-D**

…$\tilde{f}_{E\to I}(t)$ represents the *instantaneous rate of the* number of individuals becoming infectious at time $t$, $\tilde{f}_{S\to E}\left( t \right)$ denotes the *instantaneous rate of the* number of individuals exposed at time $t$,…

11. Lines 408-409: the second line of equation (18) looks potentially incorrect to me – please check.

Thank you for pointing this out. Indeed, the $\Delta E\left( t \right)$ term in the second line should be $-\Delta E\left( t \right)$. This was a simple typo; all simulations were performed with the correct equation. We have revised the typo in the Manuscript.

**Methods:**

**Construction and simulation of the SEIR model with Gamma-distributed latent and infectious periods**

$$f_{S\to E}\left( t \right)=-\Delta S\left( t \right)=-\left( S\left( t \right)-S\left( t-1 \right) \right)$$

$$f_{E\to I}\left( t \right)=-\Delta E\left( t \right)+f_{S\to E}\left( t \right)=-\left( E\left( t \right)-E\left( t-1 \right) \right)-\left( S\left( t \right)-S\left( t-1 \right) \right)$$

(18)

12. Caption to Fig S1: should (e.g.) log2(𝐸1/𝐸0) = 22.5 be 𝐸1/𝐸0 = 22.5 (or equivalently, log2(𝐸1/𝐸0) = 2.5)?

Yes. We have revised this typo.

**Supplementary Information:**

**S1 Fig** …When $\mathfrak{R}=2$ (red curve), $E_{1}/E_{0}=2^{2.5}$ led to a 37.9% relative error and $E_{1}/E_{0}=2^{-2.5}$ led to a 19.4% relative error. When $\mathfrak{R}=4$ (blue curve), $E_{1}/E_{0}=2^{2.5}$ led to a 42.5% relative error and $E_{1}/E_{0}=2^{-2.5}$ led to a 34.1% relative error.

13. Lines 424-425: is it possible to produce a plot comparing the incidence data in Fig. 4a with an equivalent version using all notified cases (not just those with available contact and symptom onset dates), to confirm there’s no substantial bias arising through a time-varying rate of contact/symptom data collection?

We compared the incidence data in Fig 4a with an equivalent version calculated from all notified cases with recorded symptom onset dates (Fig. R3). The two sets of incidence data exhibit similar trends and are highly correlated, with a Pearson correlation coefficient of 0.89. This suggests that the time-varying rates of contact and symptom onset data collection did not introduce substantial bias.

**Fig. R3 Incidence data were not influenced by the time-varying rates of contact date and symptom onset date.** The graph comparing the incidence data derived from all notified cases with symptom onset dates (blue) and a subset of cases with both contact and symptom onset dates (red; same as in Fig 4a), normalized by their respective maxima. The two sets of incidence data exhibit a similar trend, indicating that the time-varying rates of contact date and symptom onset date did not significantly affect the overall incidence pattern.

14. Lines 429-430: how did you calculate the value of E at the first time point?

While Fig 4 highlighted the performance of Hist-D during the period from August 13th, 2020 to November 25th, 2020—capturing both the growth and decline phases of the exposed population—the COVID-19 data used in this study recorded the cases starting from January 20, 2020, the date of the first officially confirmed COVID-19 case in Seoul. We assumed that the value of E was zero on this date (i.e., January 20, 2020), then calculated the value of E at the first time point shown in Fig 4 (i.e., August 13th, 2020) by cumulatively summing the difference between the daily incidence of becoming infectious and the daily incidence of exposure. We have clarified this point in the revised manuscript and apologize for any confusion caused by the earlier lack of explanation.

**Methods:**

**Data collection and preprocessing**

For the real-world data analysis, we utilized contact tracing data of COVID-19 in Seoul, Republic of Korea from *January 20^th^, 2020* to November 25^th,^ 2020. *From this data, the period from August 13^th^, 2020 to November 25^th,^ 2020* was chosen as the testing set for Hist-D and Hist-I as it includes both the increasing phase and decreasing phase of exposed individuals….

…Then, we calculated the population in the E compartment *by setting* $E\left( t_{0} \right)=0$ *on January 20^th^, 2020, the date of the first officially confirmed COVID-19 case in Seoul (18), and cumulatively summing* the difference of $f_{S\to E}$ and $f_{E\to I}$. The resulting values were used as true values of $E\left( t_{0} \right)$.

**Reviewer #2:**

This is an interesting paper that introduces a new method for estimating the initial condition of the exposed (E) class in an SEIR epidemic model that considers exposure history. Conventional methods do not account for different timing of exposure among individuals in compartment E, and the main results of this paper compare the improved accuracy of the history-dependent method to the conventional (history-independent) method. The authors apply their method to COVID-19 data from Seoul, South Korea during the first year of the pandemic, and they show that their method significantly reduces the estimation error compared to the conventional method. This is an original and important contribution to the field and the methodology is rigorously developed. The authors also provide a well documented R package to run simulations and to facilitate the use of their method. The manuscript is well written and well-organized. I only have minor comments and a few typos to fix. I recommend publication with minor revision, as detailed below.

Major:

1. My main comment is about the motivation for why the authors consider a history-dependent approach for initial condition estimation. Specifically, the first paragraph of the Results section could be expanded to include more detail and the motivation could be made more clear. One idea is to move or summarize some of the information from the Supplement (Fig 1 and Note 2) to this paragraph. Or a small table in the main text here could give a better interpretation of Supplementary Fig 1 and Note 2 for a few different scenarios (% reduced or increased initial condition and resulting error). While reading this first paragrah in the Results, I found myself paging back through the Supplementary info and it broke up the flow of the paper for me. Adding a little more motivating details and justification here will greatly improve readability. Also, it wasn’t clear where the 33.9% relative error cited in this paragraph came from... it wasn’t easy to quickly skim the Supplementary info and figure that out. Consider making this easier to decipher for the reader.

We thank the reviewer for this insightful comment, which has helped improve the clarity and value of our manuscript. As suggested, we have expanded the explanation of S1 Fig (i.e., Supplementary Fig. 1 in the original manuscript) to better highlight the motivation for accurately estimating the initial condition. Specifically, mathematical models, even with identical parameter values, can yield different simulation results depending on their initial conditions. Consequently, using different initial conditions to fit the same model to identical data can result in different estimates of key parameters, such as the reproduction number. As an illustrative example, S1 Fig demonstrates how variations in the initial condition of E in the SEIR model lead to differences in the estimated reproduction number. We have added this detailed explanation in the revised manuscript.

**Results:**

**The previous method is inaccurate when the latent period is non-exponential**

*Mathematical models, even with identical parameter values, can yield different simulation results depending on their initial conditions. Consequently, using different initial conditions to fit the same model to identical data also can yield different estimates of key parameters, such as* the transmission rate (β) or the reproduction number (R) in the SEIR model (Fig 1a inset).

However, we have limited the further details of S1 Fig in the main text, as reproduction number estimation is not the central focus of our study. While we agree that adding a table or detailed interpretation —as the reviewer kindly suggested— could enhance the accessibility of S1 Fig, we were concerned that doing so might shift attention away from the manuscript’s primary focus: estimating the initial condition of E. For this reason, we have summarized the key insights of S1 Fig in the main text. We hope this approach appropriately balances readability with thematic coherence, and we sincerely appreciate the reviewer’s suggestion.

2. Another main comment is that in the abstract, the authors say that applying their method to COVID-19 data from Seoul results in reducing the estimation error by 55% compared to the conventional method. However, there are two separate instances of this same claim in the text (lines 92 and 259) but the reduction in the error says 40%. I believe this is a typo. Indeed, the second one refers to Fig 4 which also seems to agree with the 55% claim. If these are not typos, please clarify why these are different.

Thank you for your careful reading and helpful comment. Indeed, the instances stating a 40% error reduction in the Introduction (line 92) and Discussion (line 259) were typos. The correct value, as shown in Fig 4 and stated in the Abstract, is a 55% reduction in estimation error compared to Hist-I. We have corrected the typos in both sections to ensure consistency throughout the manuscript.

**Introduction:**

… Furthermore, when we applied our method to real-world COVID-19 data from Seoul, South Korea, the error in initial condition estimation was reduced by *55%* compared to Hist-I. …

**Discussion:**

… Importantly, Hist-D successfully estimated the initial condition of E in real-world COVID-19 data from Seoul, Korea, reducing estimation error by *55%* compared to Hist-I (Fig 4). …

Minor:

1. Line 106: Fig 1b is mentioned first, but Fig 1a is left out of the main text discussion. This could go along with the expansion of this paragraph as described above, but at the very least consider a brief mention of Fig 1a and how to interpret the insert in the figure before discussing Fig 1b.

As suggested here and in Major Comment 1 from Reviewer #2, we have expanded the discussion related to Fig 1a.

**Results:**

**The history-independent method is inaccurate when the latent period is non-exponential**

*Mathematical models, even with identical parameter values, can yield different simulation results depending on their initial conditions. Consequently, using different initial conditions to fit the same model to identical data also can yield different estimates of key parameters, such as* the transmission rate ($\beta$) or the reproduction number ($\mathfrak{R)}$ in the SEIR model (Fig 1a inset).

2. Line 192: Add a brief summary of modifications made. This is easier for the reader than going to the supplementary info. Then, if the reader is interested they can read further.

We have added a summary of the modifications made to the modified Hist-I.

**Results:**

**The new history-dependent method outperforms the history-independent method**

This superiority of Hist-D persisted under various parameter conditions (See S1 Table) and even after modifications were made to the Hist-I method *by summing up the future daily incidence of becoming infectious*, as done in a previous study (27) (see S3 Text and S2 Fig).

3. Line 195: “Superior accuracy”- this is vague, consider adding by how much or by what range.

Thank you for the suggestion. The phrase “superior accuracy” refers to the quantitative comparison shown in Fig 3c-e, which is already discussed in the previous paragraph:

**Previous paragraph:**

As a result, Hist-D was much more accurate than Hist-I (Fig 3d), particularly *reducing the root mean squared error (RMSE) and mean absolute percentage error (MAPE) by 86% and 85%*, respectively (Fig 3e).

Given that the improvements in MAPE and RMSE are explicitly stated in the preceding paragraph, we believe further elaboration in this sentence would be redundant.

4. Lines 196-196: What exactly do you mean by ideal conditions? No measurement error? Please add a brief statement here.

We have revised the phrase as “ideal condition without measurement errors”.

**Results:**

Hist-D demonstrated superior accuracy compared to the Hist-I method under ideal conditions *without measurement errors*….

5. Lines 203-204: Why did you choose noise levels up to 0.1? Add a justification or comment here.

We reproduced the results shown in Fig 3f-h using higher noise levels—up to 0.3 in increments of 0.1. This modification did not alter the superiority of Hist-D, which consistently outperformed Hist-I across all noise levels (Fig 3f-h).

**Figures:**

**Fig 3. Hist-D outperforms Hist-I, regardless of the phase transition of epidemic dynamics and noise.** … h) The graph showing the RMSE (bars) and MAPE (line) of Hist-I and Hist-D across the different noise levels ($\sigma=0, 0.1, 0.2, 0.3$). Hist-D achieved a lower RMSE and MAPE than Hist-I across all noise levels. …

6. Lines 226-227: Consider a comment on whether the distribution of the latent period in this data looks gamma distributed.

We emphasize that Hist-D does not assume any specific parametric distribution (e.g., gamma distribution) for the latent period. Instead, Hist-D is designed to accommodate any form of latent period distribution; that is, $g_{\tau_{L}}$ in Fig 2 can represent any probability density function. This flexibility allowed us to use the empirical distribution of the latent period in our COVID-19 data analysis, thereby avoiding potential inaccuracies introduced by imposing a possibly misspecified parametric form to the latent period. We have clarified our use of the empirical distribution in the revised manuscript.

**Results:**

**Hist-D outperforms Hist-I for real-world COVID-19 data**

… Then, this real $E(t_{0})$ was compared with $E(t_{0})$ estimated by applying Hist-I and Hist-D to the $f_{E\to I}$ data and the *empirical distribution of incubation period* (Fig 4b). …

7. Line 350: Please comment on why this is a reasonable assumption for the approximation.

We thank the reviewer for this valuable comment. In our method, we approximate the daily incidence of exposure before the initial time point as a constant function to simplify the equations—specifically, equations (9) and (10) in the Methods section. Since this daily exposure function before the initial time is modulated by the latent period distribution in the equation for formulating the loss function (See equations (7) in the Methods), we expected that small deviations from the constant assumption would have a minimal impact on the estimation outcome. Indeed, despite this simplification, Hist-D accurately estimated the initial condition of E, even under non-constant epidemic growth as represented in both synthetic data (Fig 3) and COVID-19 data (Fig 4).

Nonetheless, to assess whether relaxing the constant assumption could improve Hist-D’s accuracy, we developed a new variant—Hist-D-exp—by assuming exponential growth or decay in daily exposure before the initial time, as suggested in Reviewer #1 Major Comment 2. When applied to the COVID-19 data, Hist-D-exp produced estimates merely comparable to those of Hist-D (Fig. R1). We believe this unexpected similarity in performance is likely because Hist-D-exp needs to estimate an additional parameter—the exponential growth or decay rate—which complicates the parameter optimization. For this reason, we maintained our focus on Hist-D and have discussed the limitations of the constant assumption in the revised manuscript.

**Fig. R1 Relaxing the constant exposure reaction does not enhance the performance of Hist-D.** The graph compares the true $E\left( t_{0} \right)$ (light gray bars) and estimated $E\left( t_{0} \right)$ (Hist-I: red triangles, Hist-D: green squares, Hist-D-exp: blue circles). Hist-D and Hist-D-exp yield comparable estimates of $E\left( t_{0} \right)$.

**Discussion:**

Despite the novelty of Hist-D, several limitations should be noted. First, our methods are derived from ODE-based infectious disease models, though PDE-based models are also used to account for spatial effects in disease spread (44, 45). Whether Hist-D can be extended to PDE models remains an open question, and exploring this would be a promising direction for future research. *In addition, Hist-D assumes the constant daily incidence of exposure before the initial time point (See equation (8) in the Methods section). Relaxing this assumption to accommodate scenarios such as super spreading event (46) or exponential growth (or decay) represents another important direction for future work.*

8. Lines 405-406: Please justify or comment on why you used the parameters from Refs 19 and 28.

We aimed to use parameters representative of the latent and infectious period of COVID-19. For the latent period, we adopted the parameter values from a previous study (ref. 19 in the original manuscript; ref. 23 in the revised manuscript) that fitted a gamma distribution to observed data on the COVID-19 latent period. For the infectious period, which can vary by region, we referred to another study (ref. 28 in the original manuscript; ref. 18 in the revised manuscript) that estimated the shape and scale parameters of the infectious period from the COVID-19 confirmed case data, which is identical to the COVID-19 data used in this manuscript. We have clarified this point in the Method section of the revised manuscript.

**Methods:**

**Construction and simulation of the SEIR model with Gamma-distributed latent and infectious periods**

… Additionally, we assumed that the latent period follows the Gamma distribution with shape 4.06 and scale 1.35, *based on a previous study that fitted a gamma distribution to observed data on the COVID-19 latent period (23)*. The infectious period was assumed to follow the Gamma distribution with shape 30 and scale 0.2, *as estimated in a previous study using same COVID-19 data employed in this study (18)*. …

Although we primarily used a single parameter set that best represents COVID-19 dynamics, the superiority of Hist-D is not limited to this specific condition (See S1 Table). In particular, across a range of alternative shape and scale parameters for the latent and infectious period, Hist-D consistently yielded lower RMSE and MAPE values. We have included this result in the Supporting Information.

|  | | *Infectious period* | | |
| --- | --- | --- | --- | --- |
|  |  | *(1.25, 4.8)* | *(2, 3)* | *(5, 1.2)* |
| *Latent period* | *(2, 2.5)* | *79.1%, 77.3%* | *79.2%, 77.1%* | *79.4%, 76.9%* |
|  | *(5, 1)* | *89.1%, 87.2%* | *89.2%, 87.2%* | *89.3%, 87.4%* |
|  | *(10, 0.5)* | *93.7%, 92.3%* | *93.8%, 92.3%* | *93.9%, 92.5%* |

***S1 Table. Hist-D is more accurate than Hist-I under various parameter conditions.*** *The table shows the reduction in RMSE (former) and MAPE (latter) achieved by Hist-D relative to Hist-I under the same conditions as Fig 3c–e, but with varied latent and infectious period parameters.*

9. Line 447: typo- extra comma after µk =
10. Line 471: typo- “The codes underlying this work are...”

Thank you for pointing these out. We have revised the typos.

**Reviewer #3:**

In this article, the authors address the inference of size of the latent exposed class in SEIR-like epidemic models. They contrast the bias in estimates of the size of the latent class when making the (common) assumption that the size of the initial latent class is determined by the initial size of the infected population and the expected latent period, with their preferred set of assumptions: that allows a distribution of time since infected in the exposed class, assuming that prior to the initial time point the risk of exposure (moving from S to E) is constant. The authors construct a loss function with amiltonianon (amiltonia rapid changes in the rate of exposure) and generate posterior estimates for $E(t_0)$ using amiltonian MCMC implemented in Stan, arguing that this approach is computationally efficient and results in improved estimates of the epidemic size (including latent exposures). They use both synthetic and detailed case data – that includes information on observed E(t) – for SARS-CoV-2 in South Korea to validate these results, and explore the impact of noise of different magnitude on their ability to recover the size of the exposed class.

This paper is a commendable attempt to consider the consequences of an oft used approximation for epidemic initial conditions. The detailed epidemiological case data, including exposure and onset dates, used to validate their approach is a significant strength. I have some suggestions that may help clarify the approach and the consequences of the results.

1. The adopted model structure can accommodate general distributions for the latent and infectious periods, but given for SARS-CoV-2 and other pathogens transmission prior to symptom onset is common, and capturing the generation time appropriately is important for accurate estimates of the initial and time-varying reproduction numbers, can you briefly clarify (perhaps repeating information on Hong et al. [28]?) how $g_1$ and $g_2$ are chosen to ensure an appropriate generation time in this model?

We thank the reviewer for the helpful comment. $g_{1}$ and $g_{2}$ in the equation (17), the probability density functions of the latent and infectious period, respectively, were chosen to reflect the latent and infectious period of COVID-19. Specifically, $g_{1}$ was based on the parameter estimates from a previous study (ref. 23) that fitted a gamma distribution to observed data on the COVID-19 latent period. For $g_{2}$, we referred to Hong et al. (ref. 18), which estimated the shape and scale parameters of the infectious period using the COVID-19 case data employed in this manuscript. Importantly, this choice of $g_{1}$ and $g_{2}$ successfully replicated the generation time of COVID-19 in Hong et al. (ref. 18). The rationale for these selections has been clarified in the revised manuscript.

**Methods:**

**Construction and simulation of the SEIR model with Gamma-distributed latent and infectious periods**

Additionally, we assumed that the latent period follows the Gamma distribution with shape 4.06 and scale 1.35, *based on a previous study that fitted a gamma distribution to observed data on the COVID-19 latent period (23)*. The infectious period was assumed to follow the Gamma distribution with shape 30 and scale 0.2, *as estimated in a previous study using the same COVID-19 data employed in this study (18).*

While we acknowledge the importance of generation time in reproduction number estimation, we would like to clarify that Hist-D does not estimate reproduction numbers. Instead, Hist-D is a generalizable method that estimates the initial conditions in any setting where the transition time between two compartments (e.g., latent period) is known and inflow data for the downstream compartment (e.g., daily incidence of becoming infectious) is available. In this study, we illustrated Hist-D using an SEIR model, where the upstream compartment is “Exposed” and the downstream is “Infectious” for synthetic data (Fig 3) and “Symptom onset” for real data (Fig 4). Thus, the validity of Hist-D hinges on the specification of the latent period (or incubation period) rather than the generation time. We have added this clarification to the revised manuscript to avoid potential confusion.

**Discussion:**

Although this study focused on the SEIR model, *Hist-D can be applied to any compartmental model where the transition time between two compartments is known and inflow data for the downstream compartment is available. Thus, Hist-D can be used when the daily incidence of becoming infectious and the latent period distribution are known (Fig 3), or when the daily incidence of symptom onset and the incubation period distribution are known (Fig 4).* …

2. I presume the main focus of the inference is E(t) and I(t), and can see that $g_{1,2}$ are fixed, but it would be good to be clear(er) about whether particular $\beta$ (and its evolution in fig 3i), are being inferred, and if appropriate showing some of the bivariate posterior distributions.

Our method, Hist-D, does not estimate the transmission rate ($\beta$); therefore, its posterior distribution is unavailable. The $\beta$ shown in the top panel of Fig 3i corresponds to the value used to generate the synthetic data for $f_{E\to I}\left( t \right)$ and $E\left( t \right)$, which are displayed in the middle and bottom panels of Fig 3i, respectively. We have revised the legend of Fig 3i to clarify this point.

**Figure legends:**

…(i) To assume the transition of epidemic dynamics, we abruptly changed the transmission rate, $\beta$, from $\beta_{0}$ to $\beta_{1}$ at a single point (top), *and simulated* $f_{E\to I}$ *data (middle), which were then used to* investigate the accuracy of Hist-I and Hist-D….

3. Figure 1 of the supplementary material demonstrates the bias introduced to estimates of $R_0$ generated by bias in E(t). Apologies if I have missed this in the discussion, but It would be nice to understand how this bias evolves in estimates of $R(t)$ in this modelling framework – i.e. if/when do these biases in IC become irrelevant for predicting the current model state?

As suggested, we investigated how bias in the initial condition of E evolves in estimates of time-varying reproduction number, $\mathfrak{R}\left( t \right)$ (S1 Fig, b). To do this, we simulated the SEIR model using the same parameters and initial conditions described in the S2 Text (Supplementary Note. 2 in the original manuscript). Using the simulated trajectory of R as the data, we fitted the model, estimating the transmission rate ($\hat{\beta}\left( t \right)$) and subsequently calculated the reproduction number ($\hat{\mathfrak{R}}\left( t \right)=\frac{\hat{\beta}\left( t \right)}{\gamma}\cdot\frac{S\left( t \right)}{N}$). Specifically, if we estimated $\hat{\mathfrak{R}}\left( t \right)$ at time $t$, we fitted the model to data from 10 days before $t$ (i.e., from $t-10$ to $t$) by minimizing the discrepancy between model predictions and simulated data. This procedure was repeated using different values for the initial condition of E: $\frac{E\left( 0 \right)}{4},\frac{E\left( 0 \right)}{2}, E\left( 0 \right), 2E\left( 0 \right),$and $4E\left( 0 \right)$, where $E\left( 0 \right)$ denotes the true initial value used in generating the synthetic data. The results indicate that the effect of bias in $E\left( 0 \right)$ remains for an extended duration after the time point where the initial condition was estimated and diminishes as time progresses. We have included this result in the S1 Fig.

**Results:**

**The history-independent method is inaccurate when the latent period is non-exponential**

… For example, if the initial condition for the exposed population is reduced to 25% of the original value, this leads to a 33.9% relative error in estimating the reproduction number under the parameter condition mimicking the COVID-19 dynamics, highlighting the importance of setting accurate initial conditions *(S1 Fig a). How the bias in initial conditions evolves in the estimation of the reproduction number is illustrated in S2 Text and S1 Fig b.*

**Supporting information:**

**S2 Text. Estimation of the reproduction number heavily depends on the initial conditions**

*To investigate how this bias in the initial condition of E evolves in estimates of time-varying reproduction number,* $\mathfrak{R}\left( t \right)$ *(S1 Fig), we fitted the model to the simulated trajectory of R used in S1 Fig, a, estimating the transmission rate (*$\beta\left( t \right)$*) and subsequently calculated the reproduction number (*$\mathfrak{R}\left( t \right)=\frac{\beta\left( t \right)}{\gamma}\cdot\frac{S\left( t \right)}{N}$*). Specifically, if we estimated* $\mathfrak{R}\left( t \right)$ *at time* $t$*, we fitted the model to data from 10 days before* $t$ *(i.e., from* $t-10$ *to* $t$*) by minimizing the discrepancy between model predictions and simulated data. This procedure was repeated using different values for the initial condition of* $E$*:* $\frac{E\left( 0 \right)}{4},\frac{E\left( 0 \right)}{2}, E\left( 0 \right), 2E\left( 0 \right),$ *and* $4E\left( 0 \right)$*, where* $E\left( 0 \right)$ *denotes the true initial value used in generating the synthetic data. The results indicate that the effect of bias in* $E\left( 0 \right)$ *continues for an extended duration after the time point where the initial condition was estimated and gradually diminishes over time.*

**S1 Fig. Estimation of reproduction number is dependent on the initial condition of E.** … *(b) The plot showing the estimated time-varying reproduction number ((*$\hat{\mathfrak{R}}(t)$*)) under varying levels of bias in the initial condition of* $E$*:* $\frac{E\left( 0 \right)}{4},\frac{E\left( 0 \right)}{2}, E\left( 0 \right), 2E\left( 0 \right),$ *and* $4E\left( 0 \right)$*, where* $E\left( 0 \right)$ *is the original initial condition value. The influence of bias in* $E\left( 0 \right)$ *continues for an extended duration after the time point where the initial condition was estimated, and decreases over time, leading to convergence in the estimated reproduction numbers.*

4. When discussing the limitations of the model, is it also possible that the discrepancy in coverage of CIs also due to assumption of constant exposure prior to $t_0$? E.g. Often epidemics may begin with 'super-spreading' events that the model isn't capturing (e.g. <https://www.nature.com/articles/s41467-023-42612-9>)

Thank you for the valuable comment. As the reviewer correctly pointed out, the assumption of constant exposure prior to the initial time point may not hold in scenarios involving super-spreading events, which can cause rapid growth in the early phase of an epidemic wave—an issue also raised by Reviewer #1. To account for such rapid changes in exposure, we developed a new variant of our method, Hist-D-exp, which incorporates exponential growth or decay in daily exposure before the initial time point.

Although Hist-D-exp did not yielded more accurate estimates compared to Hist-D (Please refer to our response to Reviewer #1, Major Comment 2, for the performance comparison), we further investigated whether Hist-D-exp could reduce the discrepancy in coverage of CI by accounting for additional uncertainty in exposure prior to the initial time point. To do this, we constructed CIs using Hist-D-exp, employing the same structure of likelihood and prior distributions described in Equations (19) and (20) of the Method section, with an additional normal prior on the new parameter, $\lambda$, representing the exponential growth or decay rate of exposure before the initial time.

However, this approach introduced substantial computational challenges. In particular, some credible intervals became unrealistically narrow (Fig. R4), and inference time increased dramatically (e.g., 58 seconds for Hist-D vs. 8 hours for Hist-D-exp for 9/30 in Fig. R4). We suspect these issues stem from the introduction of the new parameter $\lambda$, which resulted in a more complex posterior geometry that the NUTS sampler struggles to explore efficiently. This is evident in the convergence issues we observed, including a 100% divergent transition rate for the 9/30 in Fig. R4.

Based on these findings, we concluded that relaxing the constant exposure assumption does not necessarily resolve the discrepancies in CI coverage. Accordingly, we have therefore deferred further development of Hist-D-exp and its Bayesian inference to future work and have discussed the limitations of the constant exposure assumption in the revised manuscript.

**Fig. R4 Hist-D-exp did not reduce the discrepancy in coverages of CI.**  Comparison of 95% credible intervals and empirical coverage between Hist-D (top) and Hist-D-exp (bottom). The upper and lower horizontal lines of each green box (Hist-D) and blue box (Hist-D-exp) represent the upper and lower bounds of the credible intervals, corresponding to the 97.5% and 2.5% quantiles, respectively. 90.4% of true values were included in the 95% credible interval of Hist-D-exp, compared to 91.3% for Hist-D. Credible intervals constructed from Hist-D-exp were unrealistically narrow at some points (red arrows).

**Discussion:**

… *In addition, Hist-D assumes a constant daily incidence of exposure before the initial time point (See equation (8) in the Methods section). Relaxing this assumption to accommodate scenarios such as super spreading event (46) or exponential growth (or decay) represents another important direction for future work.*

… Additionally, the empirical coverage of the 95% credible intervals was below the expected level (i.e. 95%). This discrepancy may arise from a mismatch between the real-world data generation process and the model assumptions, *such as that constant exposure occurs before the initial point (equation (8))*, underlying the Bayesian approach.

5. Considering the version of Stan used to run the code, there was a syntax error in the data block for the current version (though easy to fix).

Thank you for pointing this out. We acknowledge that the syntax used in the data block may cause issues in recent versions of Stan. To address this, we have now specified the versions of Stan and RStan used in our analysis in the Methods section. This clarification should help ensure reproducibility and avoid compatibility issues in future use.

**Methods:**

In this study, functions to estimate the initial condition and simulate all scenarios were developed by the authors in the programming language R (version 4.3.2) and *Stan (version 2.32.2) and Rstan (version 2.32.6)*.

6. The inference did not run for me when using the input data file 'SimulData_cde.csv' due an incorrect date format. It did run as for the file specified in the code, though with many divergent transitions. Please consider checking the instructions for running the code, and reporting on the convergence diagnostics for your MCMC chains.

Thank you for your careful reading and helpful comments. As noted, the inference did not run correctly with the input data file due to an incorrect date format. We have resolved this issue by providing a new input data file with the corrected format.

Regarding the divergent transitions observed during MCMC sampling, we appreciate your observation. To address this, we increased the ‘adapt_delta’ parameter from 0.95 to 0.99 in the ‘HistD_stan’ function, which reduces the step size and encourages more conservative exploration of the posterior distribution. We also assessed convergence using the Gelman-Rubin diagnostic (R-hat), whose values below 1.1 indicate satisfactory convergence.

After these modifications, we re-estimated the initial condition for July 3, 2020, using the Hist-D package, with the updated sample input. As a result, divergent transitions were reduced to less than 0.05%, and the maximum R-hat was 1.003, confirming reliable chain convergence.
