# Supplementary material for: A history-dependent approach for accurate initial condition estimation in epidemic models
Source: PLoS Comput Biol. 2025 Sep 5;21(9):e1013438. doi: 10.1371/journal.pcbi.1013438 (PMC12445537; doi:10.1371/journal.pcbi.1013438)
Supplement: Attachment — Submitted filename: Response to reviewers_QC.docx [file pcbi.1013438.s015.docx]

We appreciate the editor’s and reviewers’ positive assessment and helpful suggestions. In response, we have made minor revisions to improve the clarity and presentation of our manuscript. Detailed responses to each comment are provided below in blue, with changes to the manuscript highlighted in green italics.

**Editor:**

Thank you for engaging so positively with the reviewers' comments. As you will see, the reviewers who had substantive comments are now almost entirely satisfied, as am I. I will leave it up to you to decide whether - or not - to address the remaining comments from Reviewer #1; despite what is said below about "only permit corrections to spelling, formatting or significant scientific errors" you should be able to address these - if you wish - as part of the process of moving to publication.

We sincerely thank the editor for the positive evaluation and guidance. Regarding the remaining comments from Reviewer #1, we have addressed those we considered helpful in enhancing the clarity and presentation of the manuscript, as detailed in our point-by-point response below.

**Reviewer #1:**

I thank the authors for their detailed responses to my comments and those of the other reviewers, and am mostly satisfied with the responses. I have just a few remaining minor comments:

We thank the reviewer for the positive assessment and helpful suggestions. We appreciate the remaining comments and have addressed all of them in the revised manuscript as detailed below.

Minor:

1. In the abstract, please modify “this history-independent method yields serious bias in the estimation of the initial condition” to “this history-independent method can yield serious bias in the estimation of the initial condition when the latent period is not exponentially distributed”. Similarly, in the author summary, change “this unrealistic assumption leads to serious errors” to “this unrealistic assumption can lead to serious errors”

We have revised the sentences as suggested by the reviewer, toning down the statements accordingly.

**Abstract:**

…Here, we show that this history-independent method *can* yield serious bias in the estimation of the initial condition. …

**Author summary:**

… However, this unrealistic assumption *can* lead to serious errors in the estimation of E(t_0_)…

2. RE my initial comment 4, I agree that the method of stages is (at least slightly) less flexible than the approach here, although it’s not quite true that the number of stages must be treated as a tuning parameter­­­­—if a parametric or empirical latent period distribution is available (as is assumed in this study), the number of stages and transition rate(s) between them could be obtained by fitting the model’s (implicit) Erlang-distributed latent period distribution to best match the ‘true’ distribution. Please revise the relevant part of the discussion accordingly.

We thank the reviewer for the clarification. As suggested, we have revised the relevant part of the discussion to note that the number of stages in the method of stages can be determined by fitting an Erlang distribution to a known empirical distribution of the latent period, rather than treating the number of stages solely as a tuning parameter.

**Methods:**

… While this can be modeled through the method of stages (15), which introduces multiple substages in the exposed compartment, it requires specifying the number of substages *by fitting an Erlang distribution to the empirical distribution of the latent period*, *which may* increase the computational cost of the optimization process. …

3. I found this sentence in the Discussion a little weird: “First, our methods are derived from ODE-based infectious disease models, though PDE-based models are also used to account for spatial effects in disease spread”, since PDE models aren’t particularly common in the (applied) modelling literature. It would be nice instead to discuss the extension of this approach to other types of models more generally (for example, stochastic compartmental models and network-based models).

We appreciate the reviewer’s helpful comment. As suggested, we have revised the discussion to remove the emphasis on PDE models and instead highlight more relevant extensions, such as stochastic compartmental models and network-based models.

**Discussion:**

… First, our methods are derived from ODE-based infectious disease models, though *stochastic compartmental models and network-based models* are also used *to better capture transmission uncertainty and detailed processes of disease progression, respectively* (46, 47). …

**Reviewer #2:**

The authors have done a nice job with the revision and have addressed all of my concerns.

We sincerely appreciate the reviewer's positive feedback on our revised manuscript. We are pleased that the modifications have successfully addressed the previous concerns and improved the paper's quality.

**Reviewer #3:**

Thank you for thoroughly responding to my comments, I am happy to recommend acceptance.

We appreciate the reviewer’s positive assessment and are pleased that our revisions have addressed the prior concerns and strengthened the manuscript.
